# Recruiting clinical personnel as research participants: a framework for assessing feasibility

**DOI:** 10.1186/1748-5908-8-125

**Published:** 2013-10-24

**Authors:** Sylvia J Hysong, Kristen Broussard Smitham, Melissa Knox, Khai-El Johnson, Richard SoRelle, Paul Haidet

**Affiliations:** 1 Center for Innovations in Quality, Effectiveness and Safety, Michael E. DeBakey VA Medical Center, 2002 Holcombe Blvd (152), Houston, TX 77030, USA; 2 Department of Medicine, Baylor College of Medicine, 2002 Holcombe Blvd (152), Houston, TX 77030, USA; 3 Department of Medicine, Meharry Medical College, Nashville, TN, USA; 4 Department of Medicine, Penn State Hershey College of Medicine, Hershey, PA, USA

## Abstract

Increasing numbers of research studies test interventions for clinicians in addition to or instead of interventions for patients. Although previous studies have enumerated barriers to patient enrolment in clinical trials, corresponding barriers have not been identified for enrolling *clinicians* as subjects. We propose a framework of metrics for evidence-based estimation of time and resources required for recruiting clinicians as research participants, and present an example from a federally funded study. Our framework proposes metrics for tracking five steps in the recruitment process: gaining entry into facilities, obtaining accurate eligibility and contact information, reaching busy clinicians, assessing willingness to participate, and scheduling participants for data collection. We analyzed recruitment records from a qualitative study exploring performance feedback at US Department of Veterans Affairs Medical Centers (VAMCs); five recruiters sought to reach two clinicians at 16 facilities for a one-hour interview. Objective metrics were calculable for all five steps; metric values varied considerably across facilities. Obtaining accurate contact information slowed down recruiting the most. We conclude that successfully recruiting even small numbers of employees requires considerable resourcefulness and more calendar time than anticipated. Our proposed framework provides an empirical basis for estimating research-recruitment timelines, planning subject-recruitment strategies, and assessing the research accessibility of clinical sites.

## Background

Federal research funding has seen an increase in research studies whose interventions for improving healthcare quality are aimed at clinicians in addition to or in lieu of patients; for example, the number of projects awarded by the National Institutes of Health focusing on providers has tripled from 2010 to 2012 [1]. Studying clinicians as the population of interest is certainly nothing new; for example, the Nurses’ Health Study [2], originally designed to investigate the potential long-term consequences of the use of oral contraceptives, has successfully followed registered nurses prospectively for over 35 years, monitoring various health-related and quality of life topics every two years. In the context of implementation research, however, clinicians serve not only as sources of data and/or the objects of intervention, but often as stakeholders and indispensable resources to ensure the success of the intervention’s implementation. Consequently, successfully recruiting clinicians in implementation studies becomes not only a matter of scientific importance, but of implementation success as well.

As clinician time becomes more precious, it becomes increasingly important for researchers to realistically gauge their access to this already stressed, vulnerable research population. Unlike the literature on patient recruitment, which has a rich, diverse knowledgebase of hundreds of studies, research on recruiting clinicians as research subjects is fragmented and scarce. For example, common strategies for maximizing recruitment of clinicians include minimizing respondent burden, sizeable incentives, capitalizing on existing relationships, and personal contact with target subjects; such strategies are drawn from papers examining different subject populations, such as individual physicians, individual nurses, and entire medical practices [3-5]. Consistent with some of these strategies, Solberg and colleagues [6] proposed seven factors researchers must mind to maximize participation by entire medical groups, including longstanding relationships with the target clinicians, an established reputation, and rewards; however, achieving the 100% participation rate they reported required herculean efforts not often included in the timelines or budgets of research proposals. Other factors that have been noted to impact clinician recruitment include less obvious matters, such as institutional affiliation [6], clinical setting [7], and delays in Institutional Review Board (IRB) approvals [8].

The aforementioned factors, while important and effective, provide uncoordinated guidance to help design a successful recruitment campaign, and even less help in estimating the time and resources required. Recently, Broyles and colleagues noted that most published studies using clinicians as subjects (especially nurses) 'provide little information regarding recruitment strategies, recruitment rates, participation rates, and representativeness rates’ [3]. It is not surprising, then, that researchers often design their clinician recruitment strategy heuristically, or that reviewers often rely on personal experience to judge the adequacy of a research proposal’s recruitment plan. This could lead to considerable deviations from originally proposed timelines and budgets, potentially resulting in wasted effort and research dollars.

Recruiting clinicians as participants often takes far longer than anticipated, adversely affecting timelines, budgets, and, potentially, research findings; barriers exist at all points in recruitment, including: gaining entry into facilities, obtaining accurate eligibility and contact information, reaching busy clinicians, persuading eligible candidates to participate (without coercion), and scheduling willing participants for data collection.

Although previous research of patient recruitment has identified numerous barriers to patient enrolment in clinical trials, including physician time restrictions and clinical trial awareness [9-11], corresponding barriers have not been identified for enrolling clinicians as subjects. More importantly, we are unaware of any existing metrics to guide investigators in research planning. Consequently, our objective is to propose a framework of metrics for estimating time and resources required for recruiting clinicians as research participants in clinical studies; as an illustrative example, we present initial estimates from a federally funded study recruiting clinicians at 16 sites. With this framework, we aim to provide an evidence-based means of estimating and evaluating the feasibility of clinician-recruitment strategies in funded research.

## Method

### Proposed framework

Normally, recruitment is a single line in a timetable for a research project. A more detailed, task-based approach is warranted to accurately estimate recruitment time and resources needed. The process map methodology [12] is commonly used in work analysis as well as in Lean processes to identify both the universe of activities in a work process and critical barriers to efficient and effective outcomes. Thus, to accomplish our goal, we created a process map of our recruitment approach, and identified five basic activities for which metrics could be devised and time estimates calculated: gaining entry; obtaining accurate records; reaching participants; assessing willingness to participate; and scheduling participants for the desired procedure. Table 1 describes the specific metrics we formulated for each of these recruitment tasks. Although recruitment tactics may vary from project to project, we propose that these five basic activities are applicable to recruitment approaches for a wide variety of research designs.

**Table 1:** Metrics quantifying efforts to recruit clinicians for a one-hour telephone interview

**Step**	**Metric**	**Mean or %**	**SD**	**Min-max**	**n**
**Gaining entry**	1. Number of contact attempts to site needed to establish authorization to recruit at a given site.	6.4	8.0	1–27	16
**Obtaining accurate records (Figure**1**)**	2. Percent of potential participants assumed eligible from employee database queries who were actually ineligible to participate ('dead ends’).	67% (148/221)	-	-	-
**Reaching participants**	3. Number of contact attempts to a potential participant prior to receiving an initial response.	3.6	3.5	1–18	68
	4. Cycle time in calendar days from initial contact attempt to initial participant response.	9.5	9.9	0–50	68
**Assessing willingness to participate (Figure**1**)**	5. Percent of potential participants who declined (of those who responded to recruiter inquiry and were not lost to contact or ineligible).	53% (21/40)	-	-	-
**Scheduling participants**	6. Cycle time in calendar days from initial contact to interview completion for those agreeing to participate.	24.4	16.4	6–62	19

### Example study

To demonstrate the utility of this framework, we calculated actual metric values for each of the five tasks for a federally funded study; by calculating actual values, we not only demonstrate the utility of the framework using real data but also provide initial estimates for future researchers attempting to implement similar recruitment plans. Specific methods for the study have been published elsewhere in this journal [13]; briefly, its objective was to identify differences in the audit and feedback practices of high- versus low- and moderately performing outpatient clinics. Among other participants, we attempted to recruit one physician and one nurse at each of 16 sites for a one-hour interview, the content of which included questions about the types of quality/clinical performance information they receive and actively seek out, opinions and attitudes about the utility of said data as a form of feedback, and how they use this information.

### Participants and site selection

On the basis of filtered lists of eligible candidates, we targeted 221 primary care registered nurses and physicians at 16 geographically dispersed Veterans Affairs Medical Centers (VAMCs) for inclusion in this study, for a desired final sample of 32 clinician participants. Only full-time clinicians with at least three years in their current position were eligible.

Sites were selected using a purposive stratified approach based on their scores on a profile of 15 outpatient clinical performance measures extracted from VA’s External Peer Review Program (EPRP). We selected four high-performing, four low-performing, four consistently moderately performing (*i.e*., those with moderate average performance and the lowest variability across measures), and four highly variable facilities (*i.e*., those with moderate average performance and the highest variability across measures). For the present paper, where we explored clinician recruitment cycle times, the research team and all analyses conducted were blind to facility type; however, diverse facility performance levels ensured a broad representation of sites (see Additional file 1 for study site descriptive statistics).

### Participant recruitment

Prospective participants received an email inviting them to participate in the study and requesting a preferred contact email, phone number, and a time when a research team member can review the study information form with them and obtain informed consent. Research team members phoned the participants at their requested appointment time to review the consent form (which they received in advance of the call) and procedures with the participant, answer any questions, and schedule the interview upon agreeing to participate. Examples of the recruitment materials are presented in Additional file 2. No monetary incentives were provided for participating in the study (as are often offered in interview studies), as federal regulations prevented us from providing such incentives to federal (in this case VA) employees.

### Measures

We calculated cycle times for accomplishing each of the five recruitment tasks described earlier, using recruitment records from a qualitative study exploring performance feedback at VAMCs [14] (Table 1).

### Data sources

#### Potential participant lists

Recruiters secured filtered lists of eligible candidates from the VA’s Personnel Accounting Integrated Database (PAID); this is VA’s national employee database, which includes employment site, job title, length of employment, and full-time or part-time status (study inclusion criteria). These were used as start values for assessing task #2, obtaining accurate eligibility and contact information.

#### Contact/communications records

Five recruiters tasked to schedule two clinicians per facility for a one-hour telephone interview maintained a database of communications to sites and potential participants. The database was originally designed to track communications to potential participants for IRB auditing and documentation purposes. We queried this database to calculate number of contact attempts to site personnel and cycle times from initial contact attempt to interview completion.

## Results

**Figure 1:**
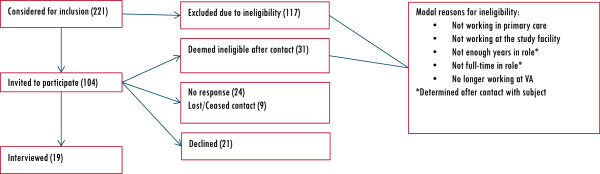
Outcomes for potential participants identified using VA PAID, filtered to eligibility requirements.

**Figure 2:**
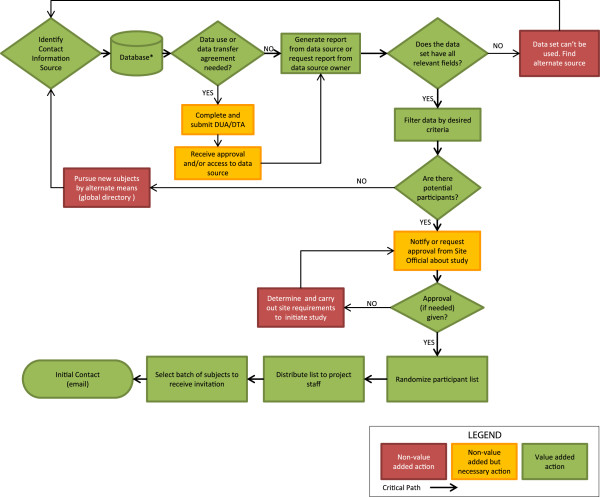
Generalized process map for obtaining accurate contact information for potential subjects.

Table 1 summarizes our observations. Table 2 provides descriptive characteristics of the clinicians interviewed. Objective metrics were calculable for all five tasks; most striking, however, was the variability in the values for each metric. For example, the standard deviations for both Reaching Participants metrics were almost equal in magnitude to the metric’s mean, indicating wide variability in cycle times and contact attempts observed. In addition, considerable variation existed across facilities in the accuracy of initial filtered lists obtained from the employee database, resulting in high percentages of ineligible prospects even after filtering lists for eligibility criteria (Figure 1). Although it was impossible to track cycle time for obtaining an accurate candidate list for a given facility with our available data sources, we interviewed the study’s recruiters to create a process map displaying all needed steps and decision points to obtain accurate contact information. Figure 2 displays a generalized map of our process for obtaining accurate contact information (Step 2 in our framework) and identifies all nonvalue-added steps. As shown, obtaining accurate contact information was much more complicated than expected. Depending on the site and its data’s accuracy and completeness, the process could be as short as seven steps (bold arrows indicate this critical path) or dramatically complicated by non-value added (black and grey boxes) actions, approvals, and error loops. Recruiters were thus forced to consult multiple sources (*e.g*., enterprise employee email directories, facility websites, clinic cold calls) to confirm eligibility (when possible) before contact, considerably delaying initial contact with the participant. Limited avenues for contact (*e.g*., no direct phone line to a clinician office) often prevented recruiters from reaching clinicians expeditiously (Table 1). Patient-care duties often interfered with clinicians’ ability to respond to recruiter communications. Lack of time to participate in an hour-long interview was the modal reason for declining our invitations.

**Table 2:** Characteristics of qualitative study interviewees

**Characteristics**	**MD (n = 12)**		**RN (n = 12)**	
	***N***	***%***	***N***	***%***
Gender				
• Male	3	25	1	8.3
• Female	9	75	11	91.7
Full time status	12	100	12	100
	***M***	***SD***	***M***	***SD***
Length of time at VA	8.7	5.3	11.7	6.9

## Discussion

This article proposes a framework for estimating time and resources required for recruiting clinicians as research participants in clinical studies, thereby providing more evidence-based means of estimating and evaluating the feasibility of clinician-recruitment strategies in funded research. Using a currently ongoing federally funded study as an example, we demonstrate our framework’s applicability to the clinician-subject population; our proposed metrics were calculable and obtainable; and, in our case, we found wide variability across study sites in the effort required to recruit clinicians. We are unaware of prior health services research studies that accomplish this objective: a PubMed search of studies examining human-subject research recruitment found no studies examining clinicians as subjects.

One of the most striking observations in the illustrative study was the high variability in cycle times, possibly a function of the facilities’ infrastructure and organizational idiosyncrasies. Because we targeted only two people at each facility, we could not calculate within-site statistics to confirm this. However, based on the content of the interviews eventually conducted for the qualitative study, we observed considerable variability in how these 16 sites operated, which could have certainly accounted for the observed cycle time variability. These observations are therefore a reminder to researchers to consider in their estimates of recruitment time the types of organizational systems that could facilitate or hinder the recruitment process.

Our findings highlight the difficulty of accessing clinicians for research purposes and the considerable barriers that exist even before the first contact attempt is made. At minimum, our study is a reminder to research teams to map their recruitment process, as well as to identify, anticipate, and prepare for potential bottlenecks. For example, site entry (Step 1) can often be a considerable barrier, especially when no a priori relationships exist between the site and the research team. Consistent with existing models of implementation [14-16], relationships with stakeholders that would normally be built as part of the implementation process could be leveraged to help gain access to both the site and to clinicians, thereby shortening cycle time. Similarly, as illustrated in Figure 2, up-to-date employee records (Step 2) could considerably shorten steps required, and therefore cycle times to initial participant contact, thus reducing wasted effort. Although the freshness of employee records is beyond the investigators’ control, facility stakeholders could advise investigators on more appropriate sources of contact information, and possibly suggest alternative means of reaching participants (*e.g*., approaching clinicians en masse as part of a regularly scheduled staff meeting).

Additionally, our study also highlights the utility of tracking participant communications throughout the recruitment and data collection process. Although not initially intended for this purpose, the example study’s communications tracker made it possible to generate estimates of time and resources for recruiting. Tracking participant communications is a practice that many studies already employ, often for legal or regulatory purposes. A few minor edits to a study’s participant communications tracking system (*e.g*., adding database fields, data entry, and/or reporting tools) could greatly facilitate participant recruitment tracking and help easily generate the metrics proposed in this study.

Our findings show initial estimates for one-hour interviews for a qualitative study; quantitative studies employing different data collection techniques such as surveys or medical chart abstraction are likely to exhibit different time estimates because of logistical concerns unique to those methods (*e.g*., online accessibility). However, the metrics proposed here are nevertheless viable and calculable for these types of methods because the initial steps of gaining entry, securing contact information, contact attempts, etc., are common to all these techniques. Just as analyses of late response and nonresponse are becoming the preferred way of assessing the quality of survey-based data [17,18], the proposed framework provides a way to estimate feasibility of work proposed in grant applications. As new, less traditional research designs emerge, perhaps new steps and metrics could be added to the framework; we consider our framework a good starting point to which future researchers can add.

### Limitations

The tools used to generate the estimates herein were not originally designed for this purpose; better estimates could be calculated had recruitment considerations been designed a priori into the example study. In addition, only recruitment challenges related to the participant-to-recruiter interaction were examined. Other types of externally imposed delays were excluded (*e.g*., IRB-related barriers [8]; shifts in organizational priorities). The potential for these to adversely impact recruiting should not be discounted.

Additionally, it is possible that the subject of our interviews may have been somewhat intimidating to participants, thereby potentially affecting recruitment success. However, the content was not deemed sensitive by our IRB; no one declined to participate due to the topic of our interviews, and only one participant requested not to be audio recorded. We therefore believe that the topic of our interview had minimal, if any impact on the cycle times observed in the example study.

More broadly, the framework is currently of limited value in that these metrics are not currently monitored or reported with currently conducted research; thus there are no existing empirical estimates of what can be considered reasonable or customary recruitment times for different types of designs (as alluded to by Broyles and colleagues) [3]. We caution that the values reported here are based on a small number of target subjects and may not be suitable as stable estimates of recruitment time; as more studies begin to monitor and report these metrics, empirical estimates can be calculated and benchmarks created for evaluating feasibility. In the meantime, our framework can be used to help guide thoughtfully designed recruitment campaigns in research proposals.

## Conclusion

Recruiting clinicians as subjects is a laborious endeavour with considerable potential impact to the implementation success of an intervention. Successfully recruiting even small numbers of employees requires considerable personnel, resourcefulness, perseverance, and more calendar time than experience and heuristics would predict. Our proposed framework and metrics provide an empirical basis for estimating research recruitment timelines, planning subject-recruitment strategies, and assessing the research accessibility of clinical sites. We encourage future researchers to report metrics such as those proposed here in their research findings so that more stable estimates of recruitment time for different study designs can be formulated.

## Competing interests

The authors declared that they have no competing interests.

## Authors’ contributions

SH is the principal investigator for the project, was responsible for the study design, and had principal writing responsibility for this manuscript. KBS, MK, KJ, and RS are project staff; they were responsible for carrying out the participant recruitment described in the study, compiling the data for and calculating the metrics described in the framework, and material edits to the manuscript. KJ is now a medical student at Meharry Medical College, but completed the contributions named above during her tenure as research staff at the Michael E. DeBakey VA Medical Center and Baylor College of Medicine. PH is the qualitative methodologist and clinician for the project. He conceptualized the idea of the feasibility framework, made material contributions to the design of the study, and made material edits to this manuscript. All authors read and approved the final manuscript.

## Supplementary Material

Additional file 1 **Descriptive statistics for study sites **[19]**.** Click here for file

Additional file 2 Participant Recruitment Materials. Click here for file
