# Supplementary material for: Recruiting clinical personnel as research participants: a framework for assessing feasibility
Source: Implement Sci. 2013 Oct 24;8:125. doi: 10.1186/1748-5908-8-125 (PMC4015152; doi:10.1186/1748-5908-8-125)
Supplement: Additional file 1 — Descriptive statistics for study sites [19]. [file 1748-5908-8-125-S1.docx]

# Additional File 1

## Descriptive statistics for study sites

| **Facility Type** | **Size (# unique patients)** | **Residents per 10k patients^†^** | **Primary Care Presence^††^** | **Number of Primary Care Personnel** |
| --- | --- | --- | --- | --- |
| HIGH | 27,222 | 0.00 | 0.12 | 35 |
|  | 49,813 | 31.42 | 0.26 | 83 |
|  | 44,114 | 0.23 | 0.37 | 56 |
|  | 27,851 | 8.62 | 0.14 | 62 |
| MODERATE: consistently average | 63,313 | 10.63 | 0.66 | 94 |
|  | 75,609 | 18.83 | 0.08 | 115 |
|  | 62,017 | 21.58 | 0.01 | 125 |
|  | 51,645 | 30.70 | 0.33 | 54 |
| MODERATE:  highly variable | 63,555 | 14.81 | 0.21 | 30 |
|  | 27,222 | 0.00 | 0.12 | 143 |
|  | 72,739 | 35.06 | 0.45 | 27 |
|  | 14,149 | 0.00 | 0.28 | 10 |
| LOW | 58,630 | 24.94 | 0.16 | 116 |
|  | 24,795 | 0.00 | 18.02 | 23 |
|  | 19,609 | 0.00 | 0.10 | 46 |
|  | 44,391 | 27.51 | 0.12 | 88 |

^†^ a measure of the strength of the facility’s academic orientation. Greater numbers mean a stronger academic orientation. 0 means no academic affiliation.

^††^ operationalized as the percent of outpatient clinic stops provided at Community Based Outpatient Clinics (a low percentage indicates the majority of outpatient/primary care occurs at the main hospital, rather than at satellite clinics)

Source: Byrne et al. 2009 [19]

Reprinted from Hysong et al., 2012 <http://www.implementationscience.com/content/7/1/45>
