# Supplementary material for: Recruiting clinical personnel as research participants: a framework for assessing feasibility
Source: Implement Sci. 2013 Oct 24;8:125. doi: 10.1186/1748-5908-8-125 (PMC4015152; doi:10.1186/1748-5908-8-125)
Supplement: Additional file 2 — Participant Recruitment Materials. [file 1748-5908-8-125-S2.docx]

# Additional File 2 – Participant Recruitment Materials

## Sample Invitation to Participate

Note: Actual names and contact information deleted for confidentiality reasons

Dear <Participant honorific and last name >:

I am pleased to invite you to participate in a research study sponsored by VA Health Services Research & Development entitled *Improving Quality of Care through Improved Audit and Feedback*.  The purpose of this study is to examine how VA Medical Centers use clinical performance information (including EPRP) to maintain and improve clinical performance at their facilities.

You were selected to participate based on your role as a clinician in a primary care clinic who receives clinical performance feedback from your facility and/or other sources, and who meets the following criteria:

- Full-time* physician in a hospital-based VAMC primary care clinic
- In position for at least three years

**Full-time is considered a minimum of 8 of 10 clinic sessions/week*

**PLEASE RESPOND**

In the next few days, <study team member> will contact you to answer any questions you might have and to set up a convenient interview appointment.  You may contact him at [<study](mailto:richard.sorelle@va.gov) team member e-mail> or <study team member phone number.  You may also contact the study coordinator, <coordinator name>, at<coordinator phone> or [<coordinator](mailto:melissa.knox@va.gov) e-mail>.

**YOUR PARTICIPATION**

If you agree to take part in this research, your participation will consist of a telephone interview (approximately 60 minutes) and a review of the attached information sheet (approximately 10 minutes).  During the interview, you will be asked about the types of clinical performance information provided at your facility, how this information is used to improve clinical performance, and any other information you think will add to our understanding of clinical performance feedback at your facility.

As one of several participants from the <facility name>, you will be part of a study that includes 15 other VA facilities. Your participation in the study and your responses will remain confidential; all study reports will be presented in aggregate, thus no individual facility or participant will be identified. The attached study information sheet contains confidentiality procedures as well as further details about the study.

We have notified <Research Director Name>, your ACOS of Research, about the study, which is approved by our Institutional Review Board of record, Baylor College of Medicine.

I hope that you will consider taking part in this research. Your participation will contribute to our understanding of clinical performance feedback at VA Medical Centers across the country.

Thank you in advance for your consideration!

**Sylvia J. Hysong, Ph.D.***Health Services Researcher
Director, PhD Post-Doctoral Fellowship Program in Health Services Research*

Houston VA Health Services Research and Development Center of Excellence
Michael E. DeBakey VA Medical Center
[http://www.hsrd.houston.med.va.gov](http://www.hsrd.houston.med.va.gov/)

*Assistant Professor of Medicine*Baylor College of Medicine

## Sample Study Information Sheet (for Informed Consent)

The following information was provided to the participant in advance of a telephone appointment to review consent procedures with the participant. Although we gave participants the option of making the consent process and the interview separate telephone calls, most participants elected to be interviewed immediately after informed consent was obtained.

**IMPROVING QUALITY OF CARE THROUGH IMPROVED AUDIT AND FEEDBACK:
THE ROLE OF LEADERSHIP**

**Sponsor:** Veterans Affairs Health Services Research & Development (VAHSR&D)

**Principal Investigator:** Sylvia J. Hysong, PhD, Houston VA HSR&D Center of Excellence, Michael E. DeBakey VAMC (Houston)

**Baylor College of Medicine (Michael E. DeBakey VAMC) IRB Protocol Number:** H-20386 **Introduction**

You are invited to take part in an interview for a research study. Please read the following information and feel free to ask any questions before you agree to take part in the study.

A chief purpose of the VA's External Peer Review Program (EPRP) is to provide a nationwide, integrated performance management system that provides medical centers and outpatient clinics with diagnosis and procedure-specific quality of care information for use as a part of their quality management program. Feeding back the EPRP program data to the facilities and its members is a crucial step in successful quality management. We are interested in understanding how facility and network directors perceive EPRP data and use it as part of their quality management plans.

This research study is funded by VA Health Services Research and Development.

**Purpose**

The purpose of this study is to examine how leaders at VA Medical Centers use clinical performance information to maintain and improve clinical performance at their facilities. You have been asked to participate because you are in a position of leadership at your facility, or because you receive clinical performance information from your facility.

**Procedures**

Approximately 80 individuals from 16 VA Medical Centers will participate in this study. You will be one of approximately 5 individuals from your facility participating in these interviews. Your participation will consist of a telephone interview. Your interview will require about one hour and about 10 minutes for the consent process. If a follow up interview is requested, it will last no longer than 30 minutes.

A trained interviewer will ask you a set of questions about the types of clinical performance information you receive and seek out, how you use this information to improve clinical performance, and what strategies you think may be helpful for your facility or clinic to employ regarding clinical performance feedback. However you will be free to offer any relevant information.

If you decide to participate in this research study, a Houston study team member will work with you to schedule a time for the interview that is convenient for you. With your permission, the consent and interview will be digitally recorded; the interview portion will be transcribed for analysis. If you decide not to have the consent discussion and/or interview recorded, the discussion(s) will be documented via written notes.

Your participation in this study is **confidential and completely voluntary,** and your refusal to participate will involve no penalty or loss of rights to which you are entitled. You may withdraw from this study at any time without penalty or loss of VA or other benefits to which you are entitled.

The results of this study may be published or presented, but your identity and records will not be revealed unless required by Federal Law.

**Potential Risks and Discomforts**

Your participation in this study poses minimal risk to you. Participation in research may involve a loss of privacy. Because you are one of only a small number of people being invited to participate, there is a risk of loss of privacy and confidentiality. However, every effort will be made to protect your privacy and maintain the confidentiality of your responses.

Your research records will be kept as confidential as possible. All interview and consent documentation will be stored at the Houston coordinating center in double-locked storage or on a VA secured server that is located behind the VA internal firewall following VA data security guidelines. A study key linking names to code numbers will be kept in an electronic file on a secure VA server, separate from the research data. Digitally recorded Interviews will be transcribed by a professional transcribing service which has secure access to a limited section of the VA server where the files to be transcribed will be stored. All research data will be maintained in accordance with the record control schedule set out by the VA Office of Research Oversight and Office of Research and Development. Oftentimes interviews requesting candid responses about oneself can be uncomfortable or stressful. Any risk of this type of discomfort is minimal in this study, as you will be answering questions about clinical practices at your facility rather than personal information about yourself.

Although we do not foresee any social or legal risk to you, if you are a leader at your facility and/or network, you may perceive that the subjects discussed during the interview may reflect on your individual performance. Only the members of the research team and the transcriptionists will have access to your responses; your responses will be used for research purposes only and will be reported only in aggregate form. VISN and facility leadership will not have access to study data.

Study staff will update you in a timely way on any new information that may affect your decision to stay in the study.

**Potential Benefits**

You will receive no direct benefit from your participation in this study. However, your participation may help the investigators better understand how leaders at VA Medical Centers use clinical performance information to maintain and improve clinical performance at their facilities.

**Alternatives**

You may choose to not participate in this study. Participation in this interview is completely voluntary.

**Subject Costs and Payments**

You will not be asked to pay any costs related to this research. You will not be paid for taking part in this study.

**Subject's Rights**

Your consent means that you have received the information about this study and that you agree to volunteer for this research study.

You will be given a copy of this form to keep. You are not giving up any of your rights by consenting. Even after you have consented, you may change your mind at any time. Please contact the study staff if you decide to stop taking part in this study.

If you choose not to take part in the research or if you decide to stop taking part later, your benefits and services will stay the same as before this study was discussed with you. You will not lose these benefits, services, or rights.

The investigator, SYLVIA JANETTE HYSONG, and/or someone he/she appoints in his/her place will try to answer all of your questions. If you have questions or concerns at any time, or if you need to report an injury related to the research, you may speak with Melissa Knox, study coordinator, at 713-794-8611 during business hours.

Members of the Institutional Review Board for Baylor College of Medicine and Affiliated Hospitals (IRB) can also answer your questions and concerns about your rights as a research subject. The IRB office number is (713) 798-6970. Call the IRB office if you would like to speak to a person independent of the investigator and research staff for complaints about the research, if you cannot reach the research staff, or if you wish to talk to someone other than the research staff.

Under Federal Regulations, the VA Medical facility shall provide necessary medical treatment to you as a research subject injured as a result by participation in a research project approved by a VA Research and Development Committee and conducted under the supervision of one or more VA employees. This requirement does not apply to treatment for injuries that result from non-compliance by a research subject with study procedures. If you sustain an injury as a direct result of your study participation, medical care will be provided by the Michael E. DeBakey VA Medical Center. The Department of Veterans Affairs does not normally provide any other form of compensation for injury. You do not waive any liability rights for personal injury by agreeing to participate.

You may withdraw from this study at any time without penalty or loss of VA or other benefits to which you are entitled. Your participation will not affect the way you now pay for medical care at the VAMC. If you would like to verify the validity of the study and authorized contacts, you may speak with the Michael E. DeBakey Veterans Affairs Medical Center Research Office at 713-794-7918 or 713-794-7566.
